# Correlation Analysis between Traditional Chinese Medicine Syndromes and Gastrointestinal Bleeding after Percutaneous Coronary Intervention

**DOI:** 10.1155/2018/7356546

**Published:** 2018-05-31

**Authors:** Chenhao Zhang, Chaolian Huang, Mingming Wang, Xiaolin Kong, Guannan Liu, Jie Wang

**Affiliations:** ^1^Wangjing Hospital, China Academy of Chinese Medical Sciences, Beijing 100102, China; ^2^Beijing First Hospital of Integrated Chinese and Western Medicine, Beijing 100026, China; ^3^Guanganmen Hospital, China Academy of Chinese Medical Sciences, Beijing 100053, China

## Abstract

**Objective:**

To explore the characters of traditional Chinese medicine (TCM) syndromes after percutaneous coronary intervention (PCI) and to provide syndrome study theoretical evidence for TCM differentiation treatment after PCI through retrospective study.

**Methods:**

Patients with coronary heart disease (CHD) who underwent PCI in Cardiovascular Intervention Center of Wangjing Hospital during Dec. 2012 to Dec. 2014 and met the inclusion criteria were enrolled. Retrospective study was then conducted based on patients' clinical document and angiography data to explore the distribution pattern of TCM syndromes.

**Results:**

801 patients were recruited in the study. TCM syndromes in descending order of their incidence were Qi deficiency and blood stasis syndrome, heart blood stasis syndrome, Qi and Yin deficiency syndrome, phlegm and blood stasis syndrome, Qi stagnation and blood stasis syndrome, Yang asthenia syndrome, heart and kidney yin deficiency syndrome to cold congeal, and blood stasis syndrome in a more to less order. Qi deficiency and blood stasis syndrome was in the most (occurring in 298 patients, 37.20%); Qi and Yin deficiency syndrome occurred in 163 patients (20.35%); heart blood stasis syndrome was shown in 126 patients (15.73%); phlegm and blood stasis syndrome was shown in 95 patients (11.86%).

**Conclusion:**

Qi deficiency and blood stasis syndrome was closely associated with post-PCI bleeding, implying that this syndrome might serve as a powerful predictor of GI bleeding as well as a potential supplement to the current predicting and scoring system of bleeding such as CRUSADE.

## 1. Introduction

With the development of modern medical technology, percutaneous coronary intervention (PCI), which is the major therapy of coronary heart disease (CHD), has helped countless patients. However, as the opening of diseased vessel, resolving of coronary occlusion or stenosis, some postoperative adverse event badly affects the long-term safety and efficacy, making it difficult for patient to get benefit in the long run. Gastrointestinal (GI) tract bleeding after PCI, which is one of the major adverse events and has intensively threatened patients' lives, is drawing more and more attention. In traditional Chinese medicine (TCM), CHD is categorized into “chest obstruction” or “heart pain,” with the underlying etiology being “deficient origin and excessive superficiality.” Although sufficient TCM studies have been conducted focusing on analysis of PCI-related TCM syndromes, investigations aiming to analyze postoperative adverse event, especially GI bleeding, are rare, which undoubtedly impeded the utility of TCM syndrome differentiation therapy in clinical practices.

The current retrospective study enrolled 801 patients undergoing PCI and analyzed the TCM syndrome characters of patients with GI bleeding or high bleeding risk and explored the correlation between GI bleeding and TCM syndromes as well, thus providing clinical evidence for the use of TCM to prevent GI bleeding after PCI in those with high bleeding risks.

## 2. Materials and Methods

### 2.1. Patients

**Figure 1:**
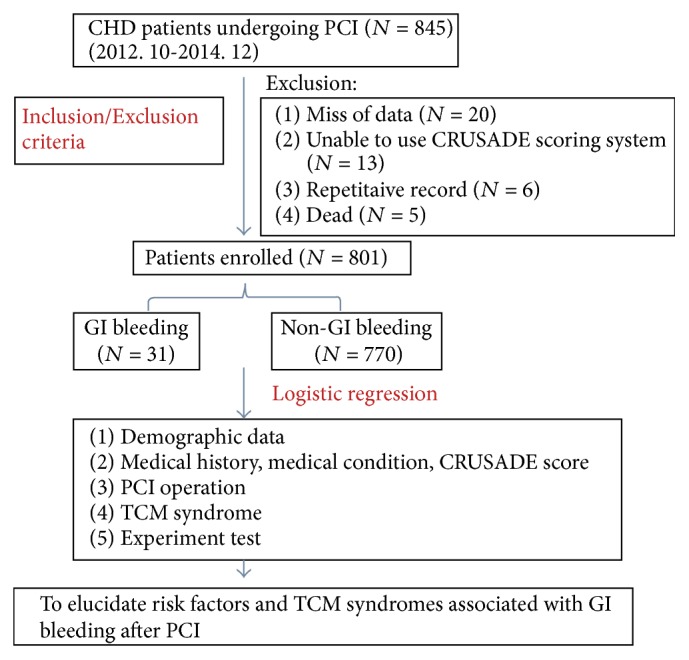
Flow chart.

845 patients undergoing PCI in Cardiovascular Intervention Center of Wangjing Hospital during Oct. 2012 to Dec. 2014 were recruited, from which 44 participants who did not meet the including criteria were excluded, and 801 participants were enrolled in the end. 31 patients reported GI bleeding and 770 are without bleeding. Retrospective analysis was performed based on participants' original clinical documents and radiography data (as shown in Figure 1).

### 2.2. Diagnostic Criteria

#### 2.2.1. Diagnostic Criteria for CHD

ST-elevated myocardial infarction (STEMI) was diagnosed according to the 2010 Guideline for the diagnosis and treatment of acute ST-elevated myocardial infarction published by Chinese Society of Cardiology [1]. Diagnosis of unstable angina pectoris (UAP) and non-ST-elevated myocardial infarction (NSTEMI) referred to 2012 Guideline for the diagnosis and treatment of non-ST-elevated myocardial infarction published by Chinese Society of Cardiology [2]. Stable angina pectoris (SAP) was diagnosed according to the 2007 Guideline for the diagnosis and treatment of chronic stable angina pectoris published by Cardiovascular Committee, China Medical Association [3].

#### 2.2.2. Diagnostic Criteria for GI Bleeding after PCI

 Recently significant hematemesis or melena and hrmatocrit (HCT) reduction by ≥15% occurred and/or hemoglobin decreased by ≥30 g/L [3].

#### 2.2.3. Criteria for Syndrome Differentiation

CHD was classified into the following eight syndromes: heart blood stasis syndrome, Qi deficiency and blood stasis syndrome, Qi stagnation and blood stasis syndrome, phlegm and blood stasis syndrome, cold congeal and blood stasis syndrome, Qi and Yin deficiency syndrome, heart and kidney yin deficiency syndrome, and Yang syndrome according to the differentiation standard for CHD angina pectoris from 〈Principles for Clinical Research of New Traditional Medicine〉 [4] revised by China Food and Drug Administration and the classification standard of chest obstruction from the national TCM higher education 12th five year version of textbook of 〈Internal Medicine of Traditional Chinese Medicine〉 [5].

### 2.3. Inclusion Criteria

1. Meeting the criteria of CHD, coronary angiography demonstrating stenosis (>70%) in at least one vessel.
2. At least one stent implantation during successful PCI operation and without operation related severe complication.
3. Full and accurate clinical document and data.

### 2.4. Exclusion Criteria

1. Severe illness in other systems, such as acute/chronic infection, blood disease, malignant neoplasm, and decompensated hepatocirrhosis.
2. Active gastrointestinal ulcer or bleeding, stomach operation within three months, cerebral hemorrhage history, recent trauma surgery, and other affecting antithrombotic drug use disorders.
3. With other severe organic cardiopathies, such as dilated cardiomyopathy, obstructive hypertrophy cardiomyopathy, and severe valvular diseases.
4. Allergic constitution and cannot accept the drugs recommended by guideline.
5. Incomplete clinical document or cannot comply with the follow-up and data collection process.

### 2.5. Operation Rules and Definition

(*1) PCI Operation Rules.* Coronary angiography (CAG) was conducted using SIMENS AXIOM ARTISDFC digital subtraction machine according to Judkins method. The regular puncture path was radial artery path, and PCI was operated according to 2005 American Heart Association Guideline for PCI. For patients without contradiction for antiplatelet agent, drug-eluting stent was recommended.

(*2) Definition for Successful PCI Operation.* CAG demonstrated significant dilation of target vessel, diameter of the rest stenosis < 20%, and coronary blood flow return to TIMI3 [6].

### 2.6. Regulation of Drug Use during Perioperative Period

Enrolled patients planning to undergo PCI should take clopidogrel of loading dose (300 mg) (Plavix, Sanofi-Aventis) and chew an aspirin (300 mg) (Aspirin, Bayer) before operation. Heparin was administrated intravenously 80~100 IU/kg at the beginning of PCI operation; low molecular weight heparin or GPIIb/IIIa receptor antagonist (Tirofiban, Yuanda Pharmaceutical, Wuhan, China) was administered electively according to the decision of operator during operation. After the operation, Tirofiban was administrated intravenously and continuously for 36–48 hours; low molecular weight heparin was injected subcutaneously for 5–7 days; aspirin (100 mg) and clopidogrel (75 mg) were taken orally; and the dose was determined by cardiologist. Antiplatelet or anticoagulation agent was withdrawn according to clinical conditions once severe bleeding occurred.

### 2.7. Regulations of Drug Use after Operation

For patients who underwent drug-eluting stent implantation, dual-antiplatelet therapy (DAPT) of aspirin (100 mg/d) and clopidogrel (75 mg/d) was required for at least one year. For those with bare-metal stent, DAPT was required for at least one month. Patients were prescribed with statins, ACEI, *β*-receptor blocker, and other drugs for secondary prevention of CHD after PCI. Risk factors including diabetes mellitus, hypertension, hyperlipidemia, and smoking were controlled.

### 2.8. CRUSADE Bleeding Score

ESC-NSTEACS recommended CRUSADE bleeding score [7] was referred to determine the classification from extremely low risk (⩽20), low risk (21~30), moderate risk (31~40), high risk (41~50), and extremely high risk (>50). Patients with CRUSADE score* ⩾* 41 were determined as high risk of bleeding.

### 2.9. BARC Bleeding Definition

Bleeding was defined according to Bleeding Academic Research Consortium (BARC) classification referred by 2011 American Academic Research Consortium (ARC) [8].

### 2.10. Research Method

Based on patients' original clinical document and angiography data, participants with GI bleeding occurrence were assigned into patient group, while those without GI bleeding were control group. Retrospective analysis was conducted to compare the demographic characters and TCM syndrome features and to explore the correlation between GI bleeding after PCI and TCM syndrome.

## 3. Clinical Data

### 3.1. General Clinical Data

Participants' age, gender, height, weight, blood pressure, and heart rate were recorded.

### 3.2. Medical History and Current Symptoms

Previous medical history (hypertension, type 2 diabetes mellitus, lipid disorder, chronic heart dysfunction, chronic kidney dysfunction, anemia, previous cerebrovascular disease, and previous peptic ulcer), smoking history, admission diagnosis (STEMI, NSTEMI, UAP, and SAP), laboratory examination (serum creatinine, creatinine clearance rate, and hematocrit), heart failure symptom, and hospital stay (days) were recorded.

### 3.3. Operation Condition

Number of stent implantation and part of diseased vessel were recorded.

*Participants' TCM symptoms, physical signs, tongue manifestation, and pulse were recorded.*

*CRUSADE score were evaluated.*

### 3.4. Bleeding (Endpoint)

The primary endpoint included incidence of GI bleeding, BARC score, and blood transfusion.

### 3.5. Data Collecting and Quality Control

In the current study, all data were obtained from the original clinical document, angiography information was exported from the catheter center of Wangjing Hospital, and clinical data acquisition was performed by physician or postgraduate of our department. Data was imported into EpiData database after check by researchers. TCM syndrome differentiation was first assessed by one experienced attending physician and confirmed by one at least associate chief physician at last. To guarantee the accuracy of data, each case was imported by at least two data administrators after consistency check and revision of any inconsistent data until the information was accurate. Two experienced physicians were designated as guide for quality control who were responsible for the supervision of data collecting process, data check, and data quality control. All information related to patients in the current study was well preserved and hided for the consideration of patients' privacy.

## 4. Statistics

### 4.1. General Principles and Methods

All statistical tests were performed using two-sided hypothesis tests at the level of *α* = 0.05 (*P* ≤ 0.05 indicates statistical significance). Specific principles are as follows:

1. Quantitative data was described with mean and standard deviation, and the hypothesis test was performed using *t*-test (normal distribution) or Wilcoxon rank-sum test.
2. Qualitative data was described with frequency and percentage, and the hypothesis test was performed using chi-square test or Fisher test. Rank data was analyzed using Wilcoxon rank-sum test.

### 4.2. Logistic Regression Model Was Applied to Analyze Risk Factors for GI Bleeding.

Logistic regression model was built with GI bleeding being primary endpoint, demographic data, medical history, and TCM syndromes being covariant. The while process was performed in a 2-step manner: firstly, single factor logistic regression analysis was performed to screen out the significant variants (*α* = 0.10). Thereafter, multiple factor regression was performed (*α* = 0.05). Maximum likelihood algorithm was used for estimation; odd ratio (OR) and 95% CI were calculated.

Software SAS9.2 was applied for data analysis.

## 5. Result

### 5.1. Demographics Characteristics

845 patients undergoing PCI from 1 Oct. 2012 to 31 Dec. 2014 were included into the current study, of which 20 patients were excluded due to miss of basic information, 13 patients were excluded due to the unavailability of CRUSADE score, 6 patients were double-recorded, and 5 were dead. As a result, 801 participants were enrolled in the study finally with the average age of 64.03 ± 12.12 years (youngest being 32 years while the oldest being 92 years), 531 males (66.29%) and 270 females (33.71%).

*Medical History.* 515 had hypertension (64.29%), 296 had diabetes mellitus (36.95%), 167 had lipid disorder (20.85%), 85 had chronic heart dysfunction (10.61%), 66 had chronic kidney dysfunction (8.24%), 16 had anemia (2%), 169 had cerebrovascular diseases (21.1%), 63 had peptic ulcer (7.87%) and 365 had smoking history (45.56%).

**Figure 2:**
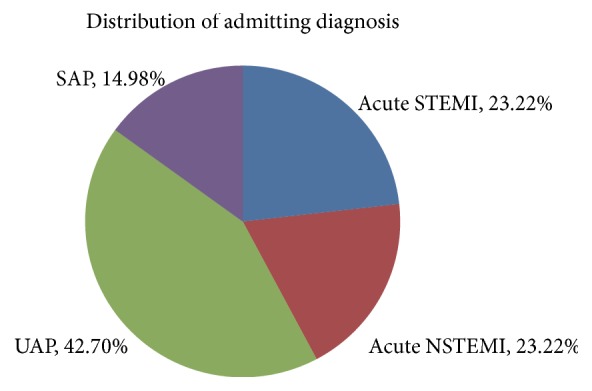
Distribution of admitting diagnosis.

*Admitting Diagnosis.* 186 had acute STEMI (23.22), 153 acute NSTEMI (19.10%), 342 unstable angina pectoris (42.70%), and 120 stable angina pectoris (14.98%). (Figure 2).

**Figure 3:**
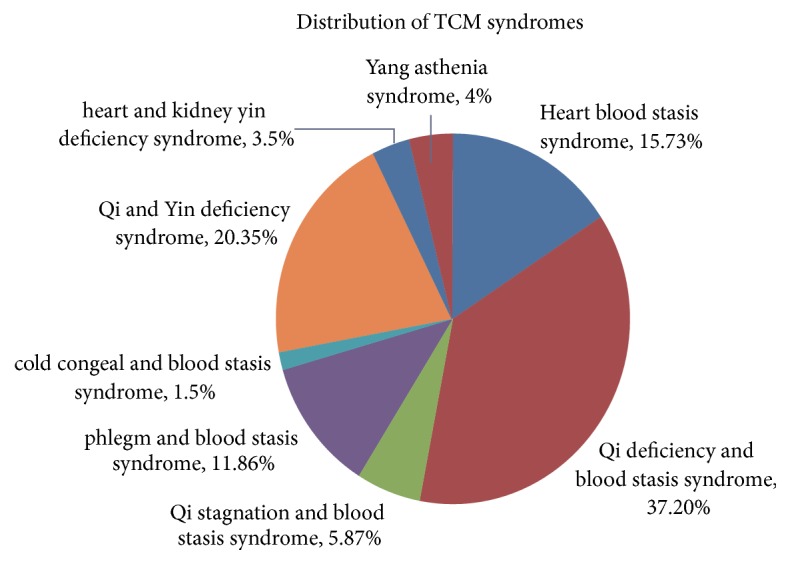
Distribution of TCM syndromes.

*Distribution Pattern of TCM Syndromes.* 126 patients presented with heart blood stasis syndrome (15.73%), 298 were Qi deficiency and blood stasis syndrome (37.20%), 47 Qi stagnation and blood stasis syndrome (5.87%), 95 phlegm and blood stasis syndrome (11.86%), 12 cold congeal and blood stasis syndrome (1.5%), 163 Qi and Yin deficiency syndrome (20.35%), 28 heart and kidney yin deficiency syndrome (3.5%), and 32 Yang asthenia syndrome (4%) (Figure 3).  Table 1 shows figures about stent implantation and Table 2 shows figures about bleeding.

### 5.2. Clinical Characters of Patients Presenting with GI Bleeding after PCI

Among the total 801 patients enrolled in the study, GI bleeding occurred in 31 participants. Significant difference could be found between GI bleeding group and non-GI bleeding group in terms of age, gender, systolic pressure, serum creatinine, creatinine clearance rate, left anterior descending (LAD) branch being diseased vessel, and hospital stay (*P* < 0.05) (Table 3).

In order to explore the correlation with GI bleeding, each predictor of CRUSADE score evaluation was analyzed. Significant difference could be found in of gender score, creatinine clearance rate score, systolic blood pressure score, CRUSADE 5 classification score, and CRUSADE 2 classification score when comparing the two groups (*P* < 0.05) (Table 4).

### 5.3. Correlation Analysis between TCM Syndromes with GI Bleeding

**Figure 4:**
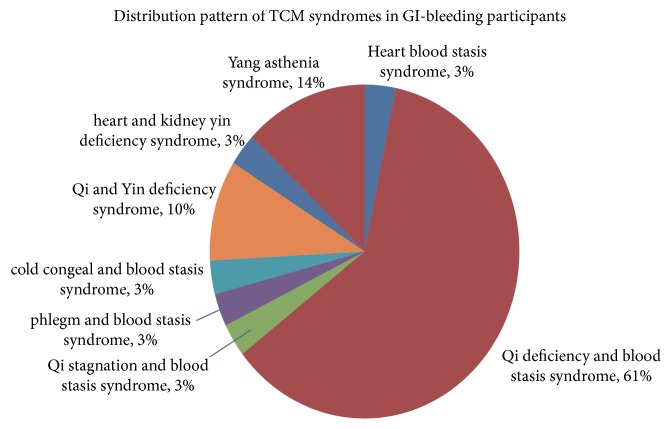
Distribution patterns of TCM syndromes in GI bleeding participants.

The distribution pattern of TCM syndromes in 31 GI bleeding participants: heart blood stasis syndrome (1), Qi deficiency and blood stasis syndrome (19), Qi stagnation and blood stasis syndrome (1), phlegm and blood stasis syndrome (1), cold congeal and blood stasis syndrome (1), Qi and Yin deficiency syndrome (3), heart and kidney yin deficiency syndrome (1), and Yang asthenia syndrome (4) (as shown in Figure 4). The results indicated significant difference in the distribution of Qi deficiency and blood stasis syndromes (*P* < 0.05) (as shown in Table 5).

Qi stagnation and blood stasis syndrome, heart blood stasis syndrome, phlegm and blood stasis syndrome, Qi and Yin deficiency syndrome, heart and kidney yin deficiency syndrome together with Yang asthenia were combined as “non-Qi deficiency and blood stasis syndrome.” We compared the incidence of GI bleeding between Qi deficiency and blood stasis group (*n* = 19) and non-Qi deficiency and blood stasis group (*n* = 12) and found statistical significant difference (*P* < 0.05) (Table 6).

### 5.4. Single Factor Regression Analysis of Risk Factors and TCM Syndromes of GI Bleeding after PCI

Single factor logistic regression model was applied to screen the risk factors and TCM syndromes of GI bleeding after PCI. To fit the regression model properly and explain the result accurately, indicators of age, BMI, and number of stent implantation were transferred into categorical data. The results indicated that indicators of age, weight, gender, CRUSADE score (5 classifications), CRUSADE (2 classifications), Qi deficiency and blood stasis syndrome, diseased vessel (left anterior descending branch), creatinine clearance rate (CRUSADE 6 classification), and systolic blood pressure (CRUSADE 5 classification) all contributed to the occurrence of GI bleeding (*α* = 0.10) (Table 7).

### 5.5. Multiple Factors Regression Analysis of Risk Factors and TCM Syndromes of GI Bleeding after PCI

Multiple factors regression analysis was conducted focusing on the predictors presenting with statistical significance: age, BMI, CRUSADE score (5 classifications), CRUSADE score (2 classifications), admitting diagnosis, TCM syndromes (2 classifications), diseased vessel (LAD), heart failure signs CRUSADE score (2 classifications), creatinine clearance rate CRUSADE score (5 classifications), and systolic blood pressure (5 classifications). The regression model indicated that CRUSADE score (5 classifications) and TCM syndromes (2 classifications) were significantly correlated with GI bleeding after PCI (*α* = 0.05).

Odd ratio (OR) of patients with extremely high score (CRUSADE score > 50) was 22.573 (95% CI: (2.976, 171.217)), indicating that the score is the most important predictor for GI bleeding after PCI. As for TCM syndromes, OR of non-Qi deficiency and blood stasis syndrome population was 0.300 (95% PCI: 0.14, 0.645), which is reverse with Qi deficiency and blood stasis syndrome group, implying that Qi deficiency and blood stasis syndrome is closely correlated with GI bleeding after PCI. Data is shown in Table 8

## 6. Discussion

The major finding in the current study is that Qi deficiency and blood stasis syndrome was closely associated with post-PCI bleeding, implying that this syndrome might serve as a powerful predictor of GI bleeding equally to age, gender, systolic blood pressure (SBP), and creatinine clearance (Ccr).

The current study analyzed the risk factors and clinical characteristics between patients with GI bleeding and without GI bleeding. As is well known, age, gender, weight, SBP, and Ccr are important predictors in CRUSADE score assessment [9–11], which is in line with the present result. As explored before, age is an evidenced predictor of bleeding. The odds ratio (OR) of predischarge major bleeding increases by 30% as ACS patients' age increases by 10 years (OR 1.28, 95% CI 1.21~1.37). With regard to gender, the incidence of bleeding complication in female is much higher than in male, possibly due to the smaller vessel, lighter weight, lower Ccr when at the same weight and blood creatinine, more comorbidities, and different pharmacological reaction when compared with males. In CRUSADE scoring system, Ccr occupies the most proportion, which means kidney dysfunction is the principle risk factors for bleeding. The underlying mechanism mainly involves the metabolite pathway of antiplatelet agents such as aspirin and clopidogrel or anticoagulation drugs such as heparin through kidney. So when the glomerular filtration rate (GFR) reduced or kidney dysfunction occurs, drugs accumulate in body, leading to increased bleeding risks.

Interestingly, our current study found that Qi deficiency and blood stasis syndrome was closely associated with GI bleeding after PCI, and this syndrome was possibly equal to CRUSADE score when serving as bleeding predictors.

### 6.1. Relation between Qi and Blood in TCM

According to TCM basic theory, the action of Qi on blood (including the controlling and leading action of Qi on blood) depends largely on governing function of spleen on blood. To be specific, when moving and generating function of spleen are vigor, both Qi and blood are simple, and thus the controlling ability of Qi is strengthened, enabling it to lead blood moving properly without oozing from vessels; conversely, if spleen is dysfunctional, source is insufficient, deficient Qi fails to control blood, leading to blood oozing out from vessels which is in other words, bleeding. Therefore, the major causation of Qi unable to control blood is dysfunction of spleen in moving and generating and incapability to lead blood. Qi failing to control blood induces blood oozing from vessel; this is blood stasis evoked by bleeding.

On other side, subsequent to bleeding, blood out of vessel together with blood despite within vessel but badly disturbed, impedes generation of new blood and induces bleeding once again; this is bleeding evoked by blood stasis, and vicious circle is initiated thereafter. The two processes always occur simultaneously and present as reciprocal causation in clinical settings. Actually, experimental researches have also reported the underlying molecular mechanism of this correlation between Qi and blood [12, 13].

### 6.2. TCM Theoretical Support for Qi Deficiency and Blood Stasis Syndrome as Post-PCI GI Bleeding Predictor

As investigators reported before, most patients with CHD presented palpitation, chest tightness, debilitation, preference to sighing, and other symptoms of Qi deficiency [14, 15] as well as blood stasis [16], either due to excessive fatigue, energy Qi consumption or feebleness with age, organ degradation, or greasy diet, spleen unable to moving and generating, moisture aggregating to phlegm, or anxiety impairing spleen inducing spleen Qi deficiency and unable to control blood. The major manifestation in clinical setting is GI bleeding such as haematemesis or melena, which is corresponding to TCM description of “deficient spleen unable to control blood and induce bleeding, and bleeding deteriorate spleen asthenia.” In addition, patient with CHD needs to lie in bed for long term to get rehabilitation after PCI operation, especially those after acute myocardial infarction attack. Lack of exercise induces less food intake, thus abatement of GI movement, dysfunction of spleen in moving and generating, reduction of absorption of essence of water and grain, and finally deficiency in Qi and blood. Simultaneously, patients always present anxiety before PCI operation, as well as depression or irritation after PCI. Heart controlled mental activities are disturbed thereafter and thus organs are jeopardized leading to deterioration of Qi deficiency.

### 6.3. Modern Investigation of the Relation between Qi Deficiency and Blood Stasis Syndrome and GI Bleeding

Many researches have been conducted to elucidate the correlation between spleen function in TCM and circulation parameters. Chaoming Zhang demonstrated that PAIgG, PAIgM, and PAIgA3 were increased significantly in purpura patients with dysfunction of spleen in governing blood, with the most evident increase in PAIgG [17], indicating that the syndrome of spleen unable to govern blood is correlated with function of platelet. Zhang et al. reported that the rosettes of C_3_b antibody in erythrocyte were the lowest in patients with syndrome of spleen unable to govern blood, implying that the immunological condition of erythrocyte was related to energy Qi in body [18]. Chen et al. demonstrated the bleeding tendency in rat with spleen Qi deficiency syndrome, and supplementing Qi and hemostasis preparation showed ideal remedial effect [19]. Wu et al. demonstrated the bleeding tendency in Qi deficiency syndrome [20], while other investigator showed the curative effect of supplementing Qi TCM formula [21].

Subsequent to the above basic findings, further investigations dedicated to illuminate the association between GI bleeding, circulation, and TCM syndromes were also reported. Fu and Jin showed that hemorheology and microcirculation abnormality could serve as the objective evidence of blood stasis syndrome, as score of erythrocyte sedimentation rate, hematocrit, plasma viscosity, blood viscosity, fibrinogen, and other microcirculation related parameters were much higher in GI bleeding group; besides, prolonged erythrocyte electrophoresis time, reduced microcirculation velocity, exudation, and bleeding were also reported, indicating that GI bleeding was closely involved with blood stasis [22]. Yonghua Shi documented the close association between blood stasis and hemorheological parameters; blood stasis acted as both result and causation of ulcer bleeding. Some activating-blood herbs could increase viscosity and hematocrit and decrease erythrocyte sediment rate, which is dual-direction regulation, which is in line with our finding.

### 6.4. Clinical Benefit

Occurrence of GI bleeding necessarily influences the overall clinical efficacy after PCI. Withdrawn or cessation of antithrombotic treatment, or even blood transfusion treatment, together with increased risks of major cardiovascular event undoubtedly prolongs hospital stays, with the increase of economic cost. In our study, patients presenting GI bleeding stayed in hospital for longer period than without bleeding, which is consistent with previous report [23, 24]. Our finding indicated that TCM syndrome differentiation might serve as a potential supplement to the current predicting and scoring system of bleeding such as CRUSADE and thus promote the endeavor in clinical settings to prevent GI bleeding after PCI. In other words, for CHD patients with this syndrome, more attention and necessary intervention should be given immediately to prevent bleeding when administering dual-antiplatelet drugs after PCI in order to obtain optimal clinical benefit.

## Figures and Tables

**Table 1:** Figures about stent implantation.

	*N* (%)
Diseased vessel	
Left anterior descending	643 (80.27)
Circumflex	436 (54.43)
Right coronary artery	519 (64.79)
Left main coronary artery	70 (8.74)
Others	80 (9.99)
Number of stent implantation	
1	289 (36.08)
2	228 (28.46)
3	127 (15.86)
4	86 (10.74)
*⩾*5	71 (8.86)
Average	2.33 ± 1.45
Total	801

**Table 2:** Figures about bleeding.

Indicator	*N* (%)
BARC bleeding grade	
1	61 (61.62)
2	25 (25.25)
3	
3a	8 (8.08)
3b	4 (4.04)
3c	1 (1.01)
GI bleeding	31 (3.87)
Blood transfusion treatment after PCI	7 (0.87)
Bleeding site	
GI	31 (31.31)
Puncture site	56 (56.57)
Urinary tract	2 (2.02)
Retroperitoneal	2 (2.02)
Cerebral hemorrhage	1 (1.01)
Gums	7 (7.07)

**Table 3:** Clinical characters of GI bleeding and non-GI bleeding groups.

Predictor	GI bleeding group (*n* = 31)	Non-GI bleeding group (*n* = 770)	*P* value
Age (years)	71.39 (±11.24)	63.74 (±12.07)	0.0005
Gender			0.0111
Male *N* (%)	14 (45.16)	517 (67.14)	
Female *N* (%)	17 (54.84)	253 (32.86)	
Weight (kg)	68.03 (±10.30)	71.39 (±11.88)	0.1537
Height (m)	1.66 (±0.08)	1.67 (±0.08)	0.5435
BMI	24.53 (±2.81)	25.47 (±3.49)	0.1361
Systolic pressure (mmHg)	123.65 (±22.07)	132.66 (±22.72)	0.0135
Heart rate (beat/min)	76.52 (±10.50)	75.44 (±14.33)	0.23
Serum creatinine (*μ*mol/L)	197.84 (±167.53)	105.55 (±105.89)	0.0006
Creatinine clearance rate (%)	49.02 (±37.86)	83.94 (±40.44)	<0.0001
Hematocrit (%)	38.79 (±7.52)	39.64 (±5.51)	0.5460
Hospital stay (day)	17.06 (±7.92)	12.45 (±8.80)	0.0001
Smoking			0.4812
Never smoking (%)	17 (54.84)	419 (54.42)	
Smoking *N* (%)	13 (41.94)	341 (44.29)	
Previous smoking *N* (%)	1 (3.23)	10 (1.30)	
Medical history			
Hypertension	18 (58.06)	497 (64.55)	0.4603
Type 2 diabetes mellitus	11 (35.48)	285 (37.01)	0.8627
Lipid disorder	5 (16.13)	162 (21.04)	0.5094
Chronic heart dysfunction	2 (6.45)	83 (10.78)	0.7636
Chronic kidney dysfunction	3 (9.68)	63 (8.18)	0.7358
Anemia	0 (0.00)	16 (2.08)	1.0000
Cerebrovascular disease history	9 (29.03)	160 (20.78)	0.2695
Peptic ulcer	4 (12.90)	59 (7.66)	0.2964
Admitting diagnosis			0.2789
STEMI *N* (%)	10 (32.26)	176 (22.86)	
NSTEMI *N* (%)	8 (25.81)	145 (18.83)	
UAP *N* (%)	11 (35.48)	331 (42.99)	
SAP *N* (%)	2 (6.45)	118 (15.32)	
Diseased vessel			
Left anterior descending branch	29 (93.55)	614 (79.74)	0.0482
Circumflex branch	20 (64.52)	416 (54.03)	0.2502
Right coronary	19 (61.29)	500 (64.94)	0.677
Left main coronary	3 (9.68)	67 (8.70)	0.7469
Others	4 (12.90)	76 (9.87)	0.5398
Signs of heart failure	20 (64.52)	373 (48.44)	0.0792

**Table 4:** Comparison of CRUSADE score between GI bleeding and non-GI bleeding groups.

CRUSADE score of each predictor	GI bleeding group (*n* = 31)	Non-GI bleeding group (*n* = 770)	*P* value
Gender	4.39 (±4.05)	2.63 (±3.76)	0.0112
Type 2 diabetes mellitus	2.13 (±2.92)	2.22 (±2.90)	0.8632
Heart rate	1.45 (±1.39)	1.64 (±2.34)	0.3076
Signs of heart failure	4.52 (±3.40)	3.39 (±3.50)	0.0795
Perivascular disease or stroke	6.00 (±0.00)	6.00 (±0.00)	1.0000
Hematocrit	2.39 (±3.19)	1.82 (±2.60)	0.4162
Creatinine clearance rate	26.58 (±12.45)	15.64 (±11.66)	<0.0001
Systolic blood pressure	3.68 (±3.07)	2.42 (± 2.36)	0.0076
Total CRUSADE score	51.13 (±16.57)	35.76 (±16.12)	<0.0001
CRUSADE 5 classification			<0.0001
Extremely low risk *N* (%)	1 (3.23)	146 (18.96)	
Low risk *N* (%)	5 (16.13)	171 (22.21)	
Moderate risk *N* (%)	1 (3.23)	187 (24.29)	
High risk *N* (%)	3 (9.68)	108 (14.03)	
Extremely high risk *N* (%)	21 (67.74)	158 (20.52)	
CRUSADE 2 classification			<0.0001
≥41 *N* (%)	24 (77.42)	266 (34.55)	
<41 *N* (%)	7 (22.58)	504 (65.45)	

**Table 5:** Comparison of TCM syndromes between GI bleeding and non-GI bleeding groups.

TCM syndrome	GI bleeding group (*n* = 31)	Non-GI bleeding group (*n* = 770)	*P* value
Heart blood stasis	1 (3.23)	125 (16.23)	0.0627
Qi deficiency and blood stasis	19 (61.29)	279 (36.23)	0.0047
Qi stagnation and blood stasis	1 (3.23)	46 (5.97)	1.000
Phlegm and blood stasis	1 (3.23)	94 (12.21)	0.1619
Cold congeal and blood stasis	1 (3.23)	11 (1.43)	0.3794
Qi and Yin deficiency	3 (9.68)	160 (20.78)	0.1322
Heart and kidney yin deficiency	1 (3.23)	27 (3.51)	1.000
Yang asthenia	4 (12.90)	28 (3.64)	0.0509

**Table 6:** GI bleeding incidences in Qi deficiency and blood stasis syndrome and non-Qi deficiency and blood stasis syndrome.

	Qi deficiency and blood stasis syndrome *N* (%)	Non-Qi deficiency and blood stasis syndrome *N* (%)	*P* value
GI bleeding			0.0052
No *N* (%)	281 (93.67)	489 (97.60)	
Yes *N* (%)	19 (6.33)	12 (2.40)	

**Table 7:** Single factor logistic regression analysis of risk factors and TCM syndromes.

Variance	Wald					95% CI
	*β*	SE	*χ* ^2^	*P*	OR	
Age	1.4553	0.3712	15.3690	<0.0001	4.286	(2.07, 8.872)
BMI	−0.6849	0.3352	4.1759	0.0410	0.504	(0.261, 0.972)
Gender	0.9088	0.3690	6.0673	0.0138	2.481	(1.204, 5.114)
CRUSADE score, 5 classification,	0.7534	0.1692	19.8193	<0.0001	2.124	(1.525, 2.959)
CRUSADE score, 2 classifications	−1.8712	0.4362	18.4029	<0.0001	0.154	(0.065, 0.362)
Smoking	0.0541	0.3466	0.0244	0.8759	1.056	(0.535, 2.082)
History: hypertension	−0.2738	0.3717	0.5425	0.4614	0.761	(0.367, 1.576)
History: type 2 diabetes mellitus	−0.0662	0.3827	0.0299	0.8627	0.936	(0.442, 1.982)
History: lipid disorder	−0.3257	0.4962	0.4308	0.5116	0.722	(0.273, 1.91)
History: chronic heart dysfunction	−0.5606	0.7403	0.5736	0.4488	0.571	(0.134, 2.436)
History: chronic kidney dysfunction	0.1844	0.6215	0.0880	0.7667	1.202	(0.356, 4.066)
History: anemia	−12.0836	518.6	0.0005	0.9814	<0.001	(<0.001, >999.999)
History: cerebrovascular disease	0.4445	0.4055	1.2013	0.2731	1.560	(0.704, 3.453)
History: peptic ulcer	0.5796	0.5526	1.1001	0.2942	1.785	(0.604, 5.274)
Qi deficiency and blood stasis syndrome	−1.0136	0.3763	7.2567	0.0071	0.363	(0.174, 0.759)
Diseased vessel, LAD	1.3040	0.7366	3.1343	0.0767	3.684	(0.87, 15.606)
Diseased vessel, circumflex branch	0.4364	0.3823	1.3035	0.2536	1.547	(0.731, 3.273)
Diseased vessel, right coronary	−0.1567	0.3764	0.1732	0.6772	0.855	(0.409, 1.788)
Diseased vessel, left main coronary	0.1171	0.6208	0.0356	0.8504	1.124	(0.333, 3.796)
Diseased vessel, others	0.3028	0.5491	0.3040	0.5814	1.354	(0.461, 3.971)
Number of stent implantation	0.1579	0.1337	1.3943	0.2377	1.171	(0.901, 1.522)
Heart rate CRUSADE score	0.0122	0.1509	0.0065	0.9358	1.012	(0.753, 1.361)
Heart failure signs CRUSADE score	0.6602	0.3822	2.9831	0.0841	1.935	(0.915, 4.093)
Hematocrit CRUSADE score	0.1533	0.1379	1.2349	0.2665	1.166	(0.89, 1.527)
Creatinine clearance rate CRUSADE score	0.6728	0.1341	25.1778	<0.0001	1.960	(1.507, 2.549)
Systolic blood pressure CRUSADE score	0.3810	0.1383	7.5902	0.0059	1.464	(1.116, 1.92)

**Table 8:** Multiple factors logistic regression analysis of risk factors and TCM syndromes.

Variance	Wald					95% CI
	*B*	SE	*χ* ^2^	*P*	OR	
Intercept	−3.2756	1.1224	8.5163	0.0035		
CRUSADE score (5 classifications)						
Extremely high risk (>50)	3.1168	1.0338	9.0896	0.0026	22.573	(2.976, 171.217)
High risk (41~50)	1.6601	1.1673	2.0226	0.1550	5.260	(0.534, 51.835)
Moderate risk (31~40)	−0.2112	1.4200	0.0221	0.8818	0.810	(0.05, 13.091)
Low risk (21~30)	1.4968	1.1033	1.8406	0.1749	4.467	(0.514, 38.826)
TCM syndrome, 2 classifications	−1.2031	0.3902	9.5067	0.0020	0.300	(0.14, 0.645)

## Additional Points

*Limitation. *Considering the importance of bleeding definition and optimization of the clinical trial design, the current investigation applied BARC bleeding score instead of previous TIMI and GUSTO score. As the result showed, of all bleeding population, BARC 1 classification had 61 cases (61.62%); BARC 2 were 25 (25.25%); BARC 3 were 13 (13.13%). Considering the fact that TCM researches about PCI rarely use BARC score, the current data might serve as a good supplement.
